# Biodegradation mechanisms of *p*-nitrophenol and microflora dynamics in fluidized bed bioreactors

**DOI:** 10.3389/fmicb.2025.1602768

**Published:** 2025-05-14

**Authors:** Xin Zheng, Yongjie Zhang, Zhiheng Ye, Zhiyan Pan

**Affiliations:** ^1^College of Environment, Zhejiang University of Technology, Hangzhou, China; ^2^Wenzhou Center for Integrated Material and Ecological Management, Wenzhou, China

**Keywords:** *p*-nitrophenol, fluidization, hydroquinone route, biofilm, microbial community

## Abstract

*p*-Nitrophenol (PNP), a member of the nitroaromatic family, is widely used in the production of pesticides, dyes, pharmaceuticals, and petroleum products. As a toxic compound, PNP is highly resistance to degradation, posing a significant challenge in agricultural and industrial wastewater treatment. Conventional PNP wastewater treatment methods require complex operational conditions that incur high chemical and equipment costs, and potential secondary pollution. Therefore, this study developed an anoxic fluidized bed bioreactor (AFBBR) and an anaerobic-aerobic fluidized bed bioreactor (AAFBBR) to evaluate the biodegradation performance and underlying mechanisms of PNP over a period of 90 days. The effect of glucose to PNP co-substrate ratios and C/N ratios have been systemically investigated. At an influent PNP concentration of 100 mg/L, a glucose to PNP co-substrate ratio of 6:1, and a C/N ratio of 10:1, the degradation of PNP reached 88.8 ± 1.0% in the AFBBR at an HRT of 8.5 h and 95.3 ± 0.3% in the AAFBBR at an HRT of 12.7 h. Meanwhile, the mechanism of PNP biodegradation and microbial community were also studied. Results of the LC–MS/MS revealed the intermediate products and confirmed that PNP biodegradation in both reactors followed the hydroquinone as well as the hydroxyquinol pathways, with the hydroquinone pathway being dominant. Results of the 16S rRNA high throughput sequencing further revealed a predominant presence of *Proteobacteria* (34% in the AFBBR, 42 and 65% in the anaerobic as well as aerobic zones of the AAFBBR, respectively), *Firmicutes* (35, 40, and 4%), *Saccharibacteria* (14, 9, and 4%) and *Bacteroidetes* (5, 4, and 19%). In the AFBBR and the AAFBBR, the key bacterial genera responsible for PNP degradation include *Lactococcus*, *Escherichia-Shigella*, *Saccharibacteria_norank*, *Acinetobacter*, *Comamonas, Zoogloea*, and *Pseudomonas*. Notably, the hydroxyquinol pathway was observed only in the AFBBR and the aerobic zone of the AAFBBR, where *Pseudomonas* were identified as key PNP degrading bacteria. These phenomena can be attributed to the varying dissolved oxygen concentrations across different zones in the two reactors, offering valuable insights into optimizing PNP removal in pilot-scale bioreactors. This study highlights an efficient, sustainable and cost-effective approach for PNP removal from agricultural and industrial wastewater.

## 1 Introduction

As a crucial chemical compound in the nitroaromatic family, *p*-nitrophenol (PNP, C_6_H_5_NO_3_) is characterized by its toxicity, persistence, high stability and strong solubility in water (Feng et al., 2024). Meanwhile, PNP is widely used as both a synthetic intermediate or a raw material in dyes, pharmaceuticals, petroleum and pesticides (Kitagawa et al., 2004). As a result, its presence in agricultural and industrial wastewater is widespread. However, PNP pose significant threats to both ecological systems and human health since they were listed as a priority pollutant by the Environmental Protection Agency (USEPA) (Wang et al., 2024). Therefore, it is necessary to remove PNP prior to discharge of agricultural and industrial wastewater into water bodies for environmental safety and human health (Tang et al., 2012).

Traditional physical, chemical and biological techniques have been purposed for treating PNP wastewater, including adsorption, membrane filtration, distillation, oxidation, hydrogenation reduction and ion exchange (Badamasi et al., 2025). However, most of these techniques have disadvantages of complex reaction processes, high costs of chemical and equipment, and the generation of secondary pollution (Yan et al., 2022). Biological wastewater treatment (BWT) has great research and application potential in the field of organic wastewater treatment due to its sustainability and cost-effectiveness (Yan et al., 2024). Based on different aeration conditions, BWT processes are usually divided into anaerobic, anoxic, and aerobic biological treatments. Among them, a single aerobic biological treatment has the disadvantages of unstable organic carbon and nitrogen removal, as well as poor impact load resistance. Therefore, the anaerobic/anoxic biological processes are often implemented prior to aerobic processes, a configuration that has been proven effective for wastewater treatment in practical applications (Sun et al., 2025). However, due to the stable structure and biological toxicity of PNP, previous studies have found that biodegradation process is greatly inhibited during the treatment of PNP wastewater (Xu et al., 2021).

Recently, many researchers have focused on novel BWT technologies to enhance PNP degradation efficiency. The Fluidized bed bioreactor (FBBR) has been widely used for biological treatment of organic pollutants (Mallikarjuna and Dash, 2020), with advantages such as excellent mass transfer (Chowdhury and Nakhla, 2022; Lu et al., 2025), a large specific surface area (Karadag et al., 2015), high microbial activity, strong resistance to impact loads, and minimal residual biosolids production (Wang H. et al., 2020). In the FBBR, carrier particles with attached biofilm are driven by liquid or air flow to achieve a stable fluidization. By selecting various carrier particles, adjusting flow rates, and optimizing reactor structures, multiple carrier particles and diverse reaction conditions can be integrated within a single FBBR. This integration promotes the synergistic growth of functional microorganisms, enhances microbial adaptability, and improves biodegradation efficiency for wastewater treatment. Zheng et al. (2021) employed an anoxic carbon-based fluidized bed bioreactor to treat coal pyrolysis wastewater and achieved over 90% degradation of phenolic compounds with the *Brachymonas* as critical functional genus. Kuyukina et al. (2017) applied a fluidized-bed bioreactor for oilfield wastewater treatment and achieved 70% biodegradation efficiencies for alkanes and PAHs with the inoculation of co-immobilized *Rhodococcus.* In addition, Eldyasti et al. (2010) utilized a pilot-scale liquid solid circulating fluidized bed bioreactor for landfill leachate treatment, attaining COD removal efficiencies of 85%. These findings highlight the significant potential of FBBR in treating recalcitrant organic matter and persistent organic pollutants (POPs), with PNP serving as a representative example. However, most studies have focused on PNP biodegradation at the lab-scale, while research on its treatment mechanisms and microbial community composition in pilot- or full-scale reactors remains limited.

This study aimed to enhance PNP removal using the FBBR systems, indicating its biodegradation mechanisms and microflora dynamics. Stable treatment of PNP synthetic wastewater was initially achieved in an anoxic fluidized bed bioreactor (AFBBR). Subsequently, the PNP synthetic wastewater was inoculated into a pilot-scale anaerobic-aerobic fluidized bed bioreactor (AAFBBR) to further enhance the PNP removal performance. Both reactors were successfully started up and stably operated for over 90 days. Effects of glucose to PNP co-substrate ratios and C/N ratios on PNP biodegradation efficiency were tested. Intermediate products were detected to construct the PNP biodegradation mechanisms. In addition, microbial community analysis was further conducted to identify the functional bacteria within the system. In summary, this study successfully achieved enhanced PNP removal from wastewater in both the AFBBR and AAFBBR systems, and elucidated the potential biodegradation mechanisms as well as the key functional microorganisms involved in the PNP biodegradation.

## 2 Materials and methods

### 2.1 Experimental setup and operating conditions

**Figure 1:**
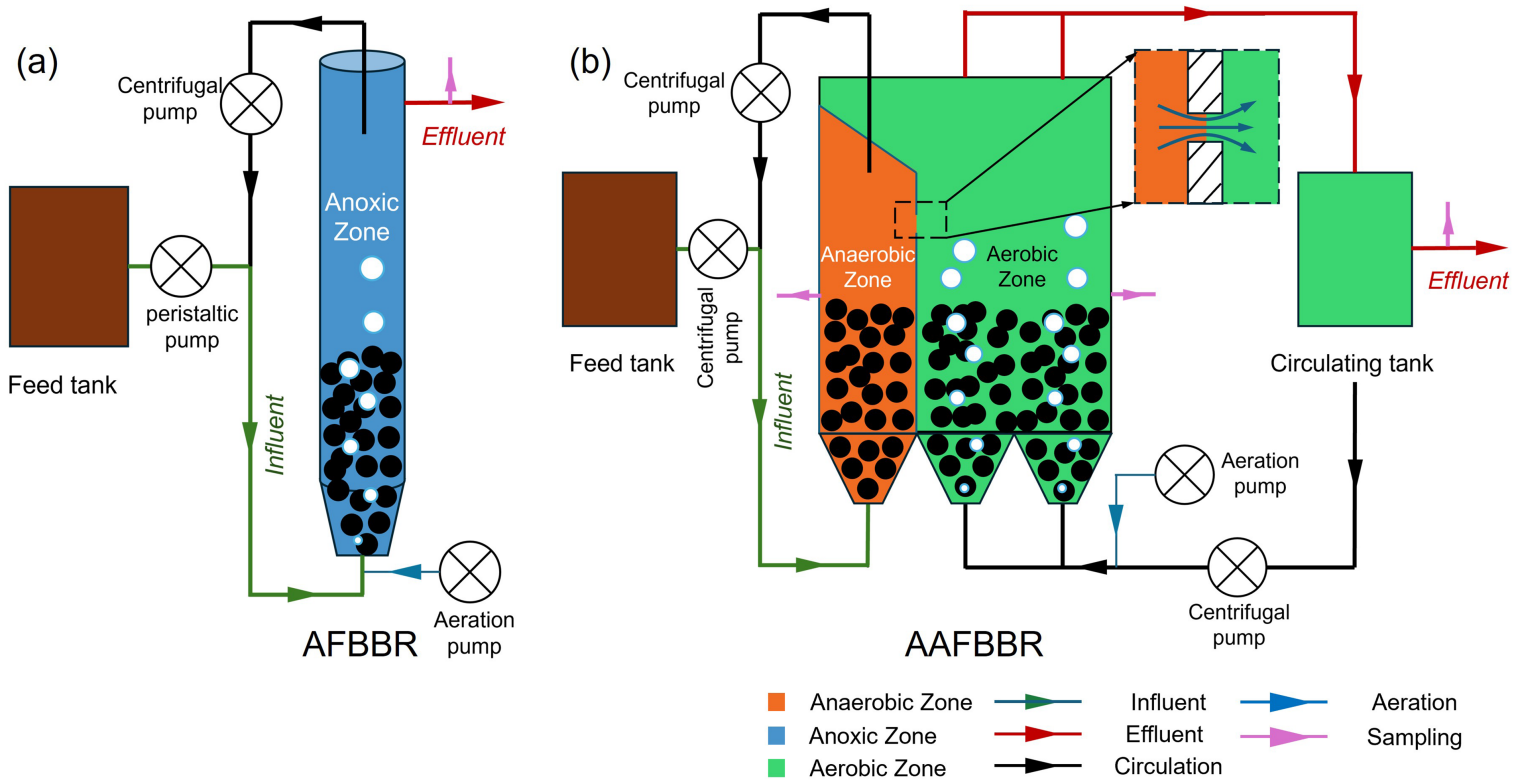
Schematic diagram of **(a)** AFBBR and **(b)** AAFBBR.

In this study, a lab-scale AFBBR and a pilot-scale AAFBBR were constructed (Figure 1), with their main bodies made of polymethyl methacrylate (PMMA). The main body of the AFBBR is cylindrical, with a height of 1,200 mm, a radius of 50 mm, and a working volume of 8.5 L. In the AAFBBR (600 mm (L) × 180 mm (W) × 1,700 mm (H)), the anaerobic and aerobic zones had working volumes of approximately 50 L and 133 L, respectively.

As shown in Figure 1a, the AFBBR was operated continuously with inlet and outlet, purging by air and controlled by ball valves to maintain the dissolved oxygen (DO) concentrations at 0.5 ± 0.2 mg/L. The internal circulating water was discharge from the top of the reactor and inoculated into the bottom by a circulating centrifugal pump (YY7112, Southern Pump Industry Co., Ltd., China) at the reflux ratios of 480:1, achieving the fluidization of the particle. Synthetic wastewater was pumped into the bottom of the reactor by a peristaltic pump (77521-50, Masterflex, United States) and then overflowed from the top. The influent flow rate was maintained at 16.2 mL/min, resulting in an HRT of approximately 8.5 h.

Similarly, as shown in Figure 1b, the AAFBBR was operated continuously with inlet and outlet. The DO concentration in the anaerobic zone was maintained at 0.0–0.2 mg/L, and the aerobic zone was purged by air and controlled by ball valves to maintain the DO concentrations between 2.0–3.0 mg/L. Particle fluidization was realized by internal circulating water driven by two circulating centrifugal pumps (YY7112, Southern Pump Industry Co., Ltd., China) at the reflux ratios of 33:1 and 139:1 in the anaerobic and aerobic zones, respectively, based on the influent flow rate of the AAFBBR. The internal circulation of the anaerobic zone was discharged from the upper part and pumped back at the bottom. In the aerobic zone, the circulating water overflowed into a circulating tank and was then pumped back into the bottom of the aerobic zone. A small channel was designed at the side of the anaerobic zone, connecting it to the aerobic zone, which allowed the wastewater to flow slowly from the anaerobic zone to the aerobic zone. Synthetic wastewater was pumped into the bottom of the anaerobic zone using an inlet centrifugal pump (AKS800NHP0800, Nanjing Suchangyuan Technology Industrial Co., Ltd., China) and flowed into the aerobic zone through the channel connecting the two zones. After microbial treatment in the aerobic zone, the wastewater naturally overflowed into the circulating tank and was ultimately overflowed from the top of the circulating tank. To facilitated sample collection, two sampling ports (as shown in Figure 1) were strategically installed in both the anaerobic and aerobic zones. The influent flow rate was maintained at 240 mL/min, resulting in an HRT of approximately 3.5 h in the anaerobic zone and 9.2 h in the aerobic zone.

### 2.2 Inoculum and synthetic wastewater composition

The initial seed sludge, with a mixed liquor volatile suspended solids concentration (MLVSS) of 4,000 mg/L, was collected from the secondary sedimentation tank of a dye manufacturing plant’s (Zhejiang Dinglong Technology Co., Ltd.) wastewater treatment facility. Having been continuously exposed to phenol-containing wastewater, its microbial community had great potential to efficiently degrade recalcitrant pollutants.

Semicokes with a particle size smaller than 3 mm are defined as waste coke particles (WCPs), accounting for approximately 15% of the total semicoke production (Hu et al., 2013). However, due to their small size, these fine semicoke particles are difficult to utilize in industries such as chemical engineering, smelting, as well as fertilizer manufacturing. and have therefore become a problematic form of solid waste (Hu et al., 2013). Nevertheless, the specific characteristics of WCPs, such as their irregular surface, large surface area, and proper density, make them highly suitable as biofilm carriers in the FBBR. Therefore, WCPs were provided by Shenmu Sanjiang Coal Chemical Co., Ltd. and utilized as carrier particles for biofilm in both reactors, with the characteristics of WCPs are listed in Supplementary Table 1.

The composition of PNP synthetic wastewater was primarily consisting of PNP, glucose, ammonium chloride and potassium dihydrogen phosphate, with NaHCO_3_ as the alkalinity source. The other constituents of the synthetic medium including trace elements are listed in Supplementary Table 2. The initial pH was adjusted to about 7.5 using either 0.1 M HCl or 0.1 M NaOH.

To ensure the stable startup and operation, 0.75 kg of WCPs were inoculated into the AFBBR, 6.75 kg and 13.50 kg of WCPs were inoculated into the anaerobic and aerobic zones of the AAFBBR, with packing ratios of 14, 20 and 13%, respectively. Additionally, 4 g, 20 g and 40 g seed sludge were inoculated into the AFBBR, as well as the anaerobic and aerobic zones of the AAFBBR, respectively. The AFBBR and the AAFBBR were then filled with PNP synthetic wastewater and continuously operated at temperature of 22 ± 2°C.

The whole experiments lasted 90 days for the AFBBR and 90 days for the AAFBBR, which were divided into three phases, summarized in Table 1: phase I involved the start-up of the PNP removal system, during which the influent PNP concentration was gradually increased from 0 to 100 mg/L to enhance the microorganisms’ tolerance capacity. In phase II, the influent glucose concentration was gradually reduced from 1,200 mg/L to 300 mg/L to achieve the different glucose to PNP co-substrate ratios, in order to investigate the effect of the glucose to PNP ratio on PNP degradation. In phase III, ammonium chloride (NH_4_Cl) was added at concentrations of 223 mg/L, 93 mg/L, 49 mg/L and 27 mg/L, achieving C/N ratios of 5:1, 10:1, 15:1, and 20:1, to examine the effect of the C/N ratio on PNP degradation.

**Table 1:** Operation conditions in the AFBBR and the AAFBBR.

Condition	Phase I	Phase II				Phase III			
	Start-up	Glucose to PNP co-substrate ratio				C/N ratio			
Time (d)	0–34	35–41	42–48	49–55	56–62	63–69	70–76	77–83	84–90
Initial NH_4_Cl (mg/L)	0	0	0	0	0	223	93	49	27
Initial PNP (mg/L)	0–100	100	100	100	100	100	100	100	100
Initial glucose (mg/L)	–	1,200	900	600	300	600	600	600	600
Temperature (°C)	22 ± 2	22 ± 2	22 ± 2	22 ± 2	22 ± 2	22 ± 2	22 ± 2	22 ± 2	22 ± 2
DO in the AFBBR (mg/L)	0.5 ± 0.2								
DO in the anaerobic zone of AAFBBR (mg/L)	0.0–0.2								
DO in the aerobic zone of AAFBBR (mg/L)	2.0–3.0								
pH	7.5 ± 0.3								

### 2.3 Analytical methods

All samples were collected and filtered through a 0.45 μm membrane filter, concentration of chemical oxygen demand (COD) and total nitrogen (TN) were measured using Chinese standard methods: the potassium dichromate oxidation method (HJ 828-2017) for COD and the alkaline potassium persulfate digestion UV spectrophotometric method (HJ 636-2012) for TN. Measurement were performed using an UV–vis spectrophotometer (UV2600, Shimadzu, Japan). DO concentration and pH were measured using a DO meter (JPB-607, REX, Shanghai, China) and precision pH meter (PHS-3Eph, REX, shanghai, China), respectively. The concentrations of PNP and *p*-aminophenol (PAP, C_6_H_7_NO) were determined following the method described by Luo et al. (2021) using high performance liquid chromatography (HPLC) with a Thermo UltiMate3000 system equipped with a C18 column (5 μm, 250 × 4.6 mm) and a UV detector. The detection wavelength was set at 254 nm. A methanol–water mixture (of volumetric proportion 40:60) was used as the mobile phase at a flow rate of 1.0 mL/min. In order to ensure the accuracy of the experiment, each test was repeated three times, and the average value was taken. The removal rates of PNP, TN, and COD in the AFBBR, as well as in the anaerobic and aerobic zones of the AAFBBR, were calculated using the following equations (as shown in Equations 1–3, with PNP removal as an example):


(1) $$\begin{array}{l} \mathrm{PNP}\;\text{removal rate in the AFBBR}\;(\%)=\\ \frac{\mathrm{Inf}.(\mathrm{PNP})-\mathrm{Eff}.(\mathrm{PNP})}{\mathrm{Inf}.(\mathrm{PNP})}\times 100\% \end{array}$$

(2) $$\begin{array}{l} \mathrm{PNP}\;\text{removal rate in the anaerobic zone of AAFBBR}\;(\%)=\\ \frac{\mathrm{Inf}.(\mathrm{PNP})-\text{anaerobic}\;(\mathrm{PNP})}{\mathrm{Inf}.(\mathrm{PNP})}\times 100\% \end{array}$$

(3) $$\begin{array}{l} \mathrm{PNP}\;\text{removal rate in the aerobic zone of AAFBBR}\;(\%)=\\ \frac{\mathrm{Inf}.(\mathrm{PNP})-\text{aerobic}\;(\mathrm{PNP})}{\mathrm{Inf}.(\mathrm{PNP})}\times 100\% \end{array}$$

To analyze the degradation intermediates of PNP, liquid chromatography-mass spectrometry (LC–MS/MS) was performed on filtered (0.22 μm organic filter membrane) samples collected from the AFBBR and the AAFBBR. Chromatographic separation was achieved using a Waters C18 column (1.7 μm × 2.1 mm, 100 mm) at 40°C, with a mobile phase consisting of 0.1% formic acid (Solvent A) and acetonitrile (Solvent B). The injection volume was 0.1 μL. Mass spectrometry was conducted in positive electrospray ionization mode (ESI+), with a drying gas flow rate of 10 mL/min, heating gas flow rate of 10 mL/min, and atomizing gas flow rate of 3 mL/min. The interface voltage was set to 4.0 kV, with an interface temperature of 30°C, a desolvation temperature of 526°C, a DL temperature of 250°C, and a heating block temperature of 400°C. Additional parameters included a conversion tap voltage of 10.0 kV, detector voltage of 1.76 kV, IG vacuum of 0.002 Pa, PG vacuum degree of 130 Pa, and CID gas pressure of 270 kPa. The scan range was set of m/z 50–500.

### 2.4 Microbial community analysis

The total genome DNA from samples was extracted using CTAB/SDS method (MK Biotechnology Co. Ltd., Hangzhou, China). The DNA concentration and purity were examined using 2% agarose gel electrophoresis. Once the concentrations of the DNA samples were recorded, DNA was diluted to 1 ng/μL using sterile water. The molecular marker chosen for this study was the 16S rRNA genes. The amplification of this marker was performed using specific primers with a barcode. The primers pairs used 16S V4-V5: 515F-907R. All polymerase chain reaction (PCR) reactions were carried out in 30 μL reactions with 15 μL of Phusion^®^High-Fidelity PCR Master Mix (New England Biolabs); 0.2 μM of forward and reverse primers, and about 10 ng template DNA. The PCR amplification conditions comprised initial denaturation at 98°C for 1 min, followed by 30 cycles of denaturation at 98°C for 10 s, annealing at 50°C for 30 s, and elongation at 72°C for 60 s. Finally, 72°C for 5 min. Sequences with ≥97% similarity was assigned to the same operational taxonomic units (OTUs).

## 3 Results and discussion

### 3.1 Reactor performance

#### 3.1.1 Effect of initial PNP concentration on PNP degradation

**Figure 2:**
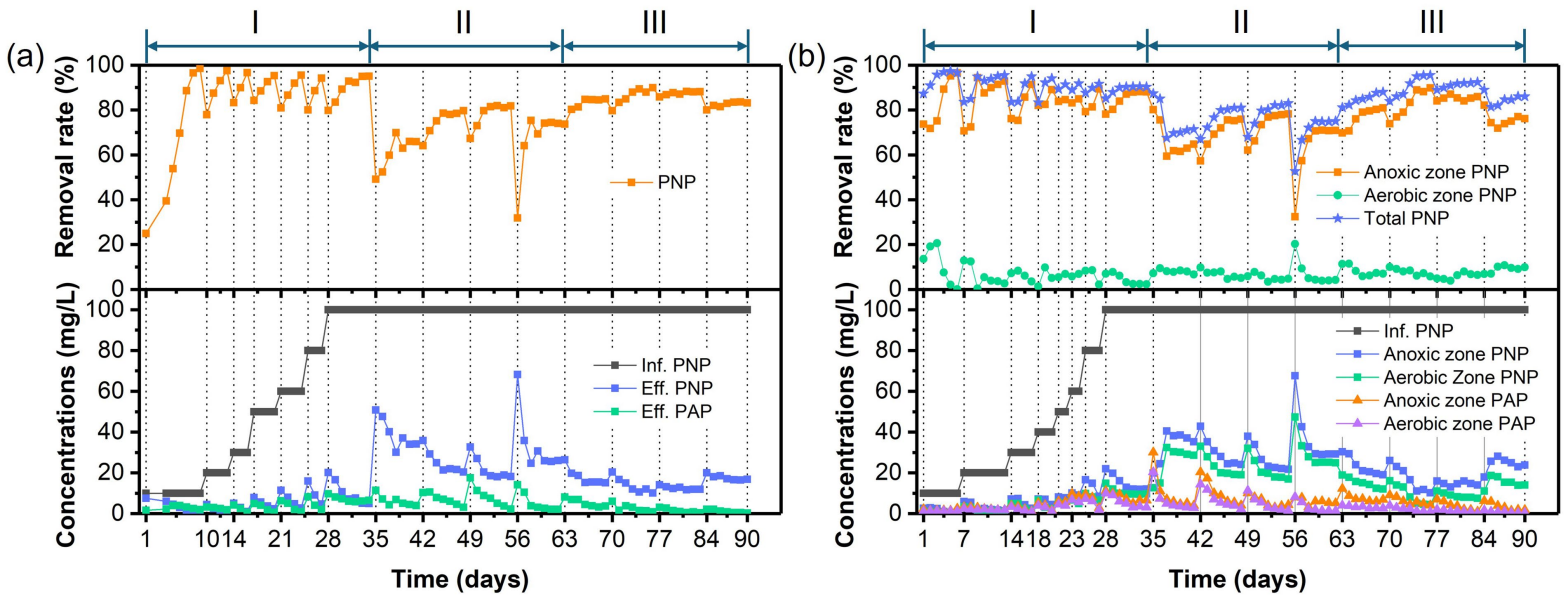
Temporal variations of PNP/PAP concentrations and removal rates in **(a)** the AFBBR and **(b)** the AAFBBRover the 90-day operation.

**Figure 3:**
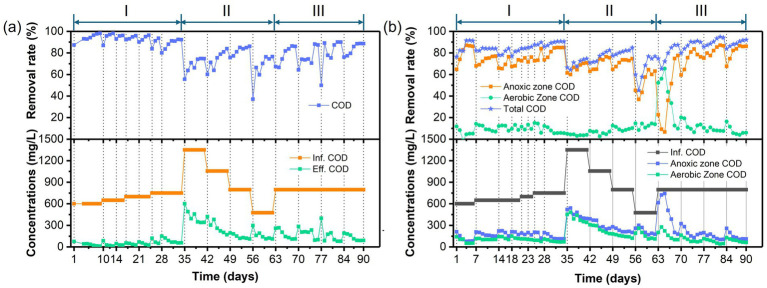
Temporal variations of COD concentrations and removal rates in **(a)** the AFBBR and **(b)** the AAFBBRover the 90-day operation.

**Figure 4:**
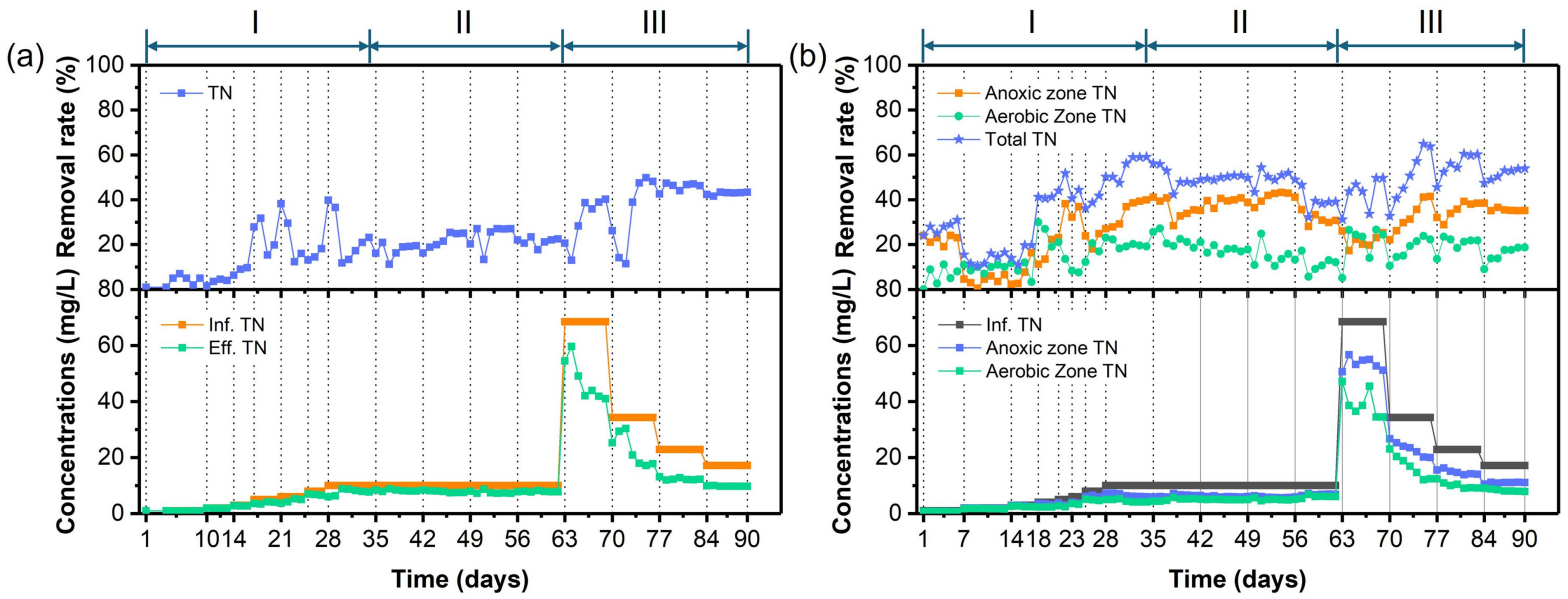
Temporal variations of TN concentrations and removal rates in **(a)** the AFBBR and **(b)** the AAFBBRover the 90-day operation.

The temporal variations in PNP, COD, and TN concentrations and removal efficiencies in the AFBBR and the AAFBBR are illustrated in Figures 2–4, respectively. In the phase I, the AFBBR and AAFBBR were performing stable COD removal efficiency over 90% at the beginning without the inoculated of PNP. Subsequently, the influent PNP concentration was gradually increased from 0 mg/L to 100 mg/L (day 1 to day 34) to realize the start-up of PNP removal. Concurrently, the influent COD concentration rose from 600 mg/L to 750 mg/L with glucose supplementation, and the influent TN concentration increased from 1 mg/L to 10 mg/L which was primarily contributed by PNP.

As shown in Figure 2, after the PNP synthetic wastewater inoculated into both reactors, AFBBR initially achieved the 25% of PNP removal efficiency and gradually increased to 90% within 7 days. While AAFBBR exhibited a strong PNP degradation capability at the beginning, achieving an initial removal efficiency of 87% on the day 1, which rose to over 90% from the second day. PNP was primarily degraded in the anaerobic zone and further broken down in the aerobic zone in the AAFBBR. The PNP removal rate in both reactors quickly exceeded 90%, attributing to the initial seed sludge which was collected from the actual wastewater treatment process of a chemical plant and exhibited excellent adaptability to phenolic wastewater. However, the AAFBBR demonstrated a faster start-up time than the AFBBR, as the synthetic wastewater first passed through the anaerobic zone, where microorganisms exhibited greater resilience to PNP, thereby mitigating its inhibitory effects on aerobic microorganisms.

During the start-up period, the AFBBR and the AAFBBR exhibited distinct response patterns to variations in influent PNP concentration, characterized by transient fluctuations and temporary declines in PNP removal efficiency following each concentration increase. In the AFBBR, the PNP removal rate dropped sharply to around 80% after each increase in PNP concentration, but recovered to above 95% within a few days. The AAFBBR showed similar fluctuations when the PNP concentration gradually increased from 0 to 40 mg/L. However, as the influent PNP concentration increased from 40 to 100 mg/L, the PNP removal rate in the AAFBBR remained at approximately 90%. This trend may be due to the initial inhibitory effects of elevated PNP concentration on the microbial community, followed by microbial adaptation and growth under the high PNP concentration (Mei et al., 2019), ultimately enhancing the PNP removal capacity in the system. The more stable PNP removal performance observed in the AAFBBR as the influent PNP increased from 40 to 100 mg/L indicated that the anaerobic microorganisms in its anaerobic zone exhibited stronger shock resistance and adaptability. While the AFBBR achieved a higher PNP removal rate than the AAFBBR (93.8 ± 1.4% vs. 90.4 ± 0.1%) at the end of phase I. This phenomenon could be attributed to the synergistic interactions among anaerobic, anoxic, and aerobic microorganisms, which played a crucial role in enhancing PNP removal efficiency in the AFBBR system. PAP is a primary reduction product of PNP under anoxic conditions (Sponza and Kuscu, 2011). However, as shown in Figure 2, PAP production rate in two reactors were lower than the reduction rate of PNP, indicating the further degradation of PAP. In the AAFBBR, the PAP concentration in the anaerobic zone was higher than that in the aerobic zone, suggesting that PAP was further degraded in aerobic zone.

Figures 3, 4 presents the temporal variations of COD and TN concentrations and removal efficiencies in AFBBR and AAFBBR. At the end of phase I, with the influent PNP concentration of 100 mg/L and the influent COD concentration of 750 mg/L, the COD removal efficiency in AFBBR and AAFBBR reached 91.8 ± 0.8% and 90.6 ± 0.1%, the TN removal efficiency in AFBBR and AAFBBR reached 20.5 ± 3.0% and 58.3 ± 1.4%, respectively. In the AFBBR, anaerobic, anoxic and aerobic microorganisms could simultaneously growth, resulting the COD removal efficiency of AFBBR is comparable to that of AAFBBR. However, biological nitrogen removal is highly dependent on DO conditions (Liu et al., 2020). In AAFBBR, the higher DO concentration in the aerobic zone created a more favorable environment for microbial nitrogen transformations, leading to a significant enhancement nitrogen removal compared to the AFBBR.

As the influent PNP concentration gradually increased, the PNP removal efficiency in both AFBBR and AAFBBR ultimately stabilized above 90% after fluctuation. At the end of phase I, the AFBBR and AAFBBR realized the PNP removal efficiency of 90.0 ± 0.1% and 93.8 ± 1.4% with the influent PNP concentration at 100 mg/L, indicating the successfully start-up of PNP removal in two reactors.

#### 3.1.2 Effect of glucose to PNP co-substrate ratio on PNP degradation

Glucose can undergo bio-oxidation to generate endogenous electron donors, which facilitate the biodegradation of organic compounds (Bai et al., 2015). In the phase II, the effect of glucose to PNP co-substrate ratio on PNP degradation was investigated in both reactors. With an influent PNP concentration of 100 mg/L, the influent glucose concentration was gradually decreased from 1,200 mg/L to 300 mg/L, corresponding to glucose to PNP co-substrate ratios of 12:1, 9:1, 6:1, 3:1.

Different glucose to PNP co-substrate ratios led microbes to utilize varying electron donors when degrading PNP through co-substrate pathways. As shown in Figure 2, decreasing the glucose to PNP co-substrate ratio in the influent from 12:1 to 6:1 led to a significant increase in PNP removal efficiency—from 65.0 ± 1.7% to 81.6 ± 0.4% in the AFBBR, and from 70.8 ± 0.7% to 82.4 ± 0.5% in the AAFBBR. The similar trend was also observed in COD removal (Figure 3). The excessive glucose supplementation negatively affected PNP degradation, which can be due to that glucose as a readily biodegradable carbon source, was preferentially consumed by microorganisms instead of PNP (Vo et al., 2020). However, further reducing the glucose to PNP co-substrate ratio to 3:1 led to a significant decline in the PNP and COD removal efficiencies in both reactors. Over the following days, as microorganisms adapted and their metabolic activity improved, the PNP removal efficiencies gradually increased and stabilized (74.2 ± 0.2% in the AFBBR and 74.9 ± 0.1% in the AAFBBR). The removal rates of PNP and COD in the two reactors remained lower than those observed when the influent glucose to PNP ratio of 6:1. These results suggest that an optimal glucose addition can enhance PNP degradation through co-substrate metabolism (Wang et al., 2022). As the influent co-substrate ratios decreased from 12:1 to 6:1, the TN removal efficiencies in AFBBR and AAFBBR kept stable (Figure 4). Further reducing the influent co-substrate ratio to 3:1, the TN removal efficiency in AAFBBR showed a significant decrease, which could be due to the limited carbon source restricted nitrogen removal through denitrification (Liu et al., 2020).

At an influent glucose to PNP co-substrate ratio of 6:1, the highest removal efficiencies of PNP, COD, and TN in AFBBR were 81.6 ± 0.4%, 85.0 ± 1.0%, and 26.7 ± 0.7%, respectively. Similarly, in AAFBBR, the maximum removal efficiencies of PNP, COD, and TN at the same glucose to PNP ratio were 82.4 ± 0.5%, 83.1 ± 1.5%, and 50.5 ± 1.6%, respectively. However, the PNP removal rates in the phase II were significantly lower than those at the end of phase I for both the AFBBR and AAFBBR. This decline was attributed to changes in influent concentration and the toxic effects of PNP, which may have led to biofilm loss.

#### 3.1.3 Effect of C/N ratio on PNP degradation

In the phase III, the effect of C/N ratio on PNP degradation in the two reactors was investigated. With the influent PNP and glucose concentration of 100 mg/L and 600 mg/L, respectively, the influent C/N ratio was systematically adjusted by supplementing nitrogen source (NH_4_Cl). C/N ratio in 5:1, 10:1, 15:1, and 20:1 was controlled with the adding of NH_4_Cl concentrations at 223 mg/L, 93 mg/L, 49 mg/L and 27 mg/L, respectively.

As shown in Figures 2, 3, the PNP and COD removal rates exhibited a significant increase in both reactors following the addition of the nitrogen source. As the influent C/N ratio increased from 5:1 to 15:1, the PNP removal performance exhibited a continuous upward trend in both reactors. In the AFBBR, the PNP removal rate increased from 84.7 ± 0.2% to 88.2 ± 0.1%, while in the AAFBBR, it increased from 87.1 ± 1.2% to 92.0 ± 0.3%. The highest PNP removal efficiency was observed at a C/N ratio of 10:1, reaching 88.8 ± 1.0% in the AFBBR and 95.3 ± 0.3% in the AAFBBR. However, further increasing the C/N ratio to 20:1 resulted in a significant decline in PNP and COD removal rates in both reactors. Given that denitrification requires organic carbon as an electron donor for nitrogen removal, the limitation of nitrogen source could reduce the organic carbon consumption and inhibit the microbial growth and metabolism (Yang et al., 2024). This trend may be attributed to the impact of the C/N ratio on bacterial extracellular polymeric substances (EPS) synthesis, both excessively high or low C/N ratio can disrupt the EPS secretion, leading to reduced biofilm formation and subsequently inhibiting PNP degradation (Ye et al., 2011). Nevertheless, even at a C/N ratio of 20:1, where the PNP removal rate declined to 83.3 ± 0.3% in the AFBBR and 85.3 ± 0.7% in the AAFBBR, the PNP removal performance in both reactors were still higher than that at the end of phase II. These findings suggest that the nitrogen supplementation effectively enhances PNP degradation in both AFBBR and AAFBBR.

As shown in Figure 4, TN removal rates exhibited a significant increase during phase III. At C/N ratio of 10:1, the maximum TN removal efficiency in the AFBBR reached 48.5 ± 1.2%, while the highest TN removal efficiency of 61.9 ± 4.2% was observed at a C/N ratio of 10:1 in the AAFBBR. The superior TN removal performance in the AAFBBR could be attributed to its integrated anaerobic and aerobic conditions, which create a more favorable DO environment for the growth and metabolism of nitrifying and denitrifying bacteria, thereby enhancing nitrogen removal efficiency (Liu et al., 2020).

### 3.2 PNP degradation mechanism in AFBBR and AAFBBR

Generally, PNP biodegradation follows two distinct pathways: the hydroquinone pathway and the hydroxyquinol pathway. In the hydroquinone pathway, PNP is sequentially transformed into benzoquinone, hydroquinone (HQ, C_6_H_6_O_2_) and γ-hydroxymuconic semialdehybe before ultimately converting into maleylacetate (Badamasi et al., 2025). In the hydroxyquinol pathway, PNP is first converted into 4-nitrocatechol (NC, C_6_H_5_NO_4_), which is further degraded into 2-hydroxy-1,4-benzoquinone and hydroxyquinol, eventually leading to the formation of maleylacetate (Badamasi et al., 2025).

On day 83, with an influent PNP concentration of 100 mg/L, a glucose to PNP co-substrate ratio of 6:1 and an influent C/N ratio of 10:1, the average PNP removal rates reached approximately 88.8% in the AFBBR and 95.3% in the AAFBBR. To elucidate the PNP degradation mechanism, intermediate products in the AFBBR, as well as the anaerobic and aerobic zones of AAFBBR, were analyzed using LC–MS/MS.

Supplementary Table 3 presents the primary intermediate products in the AFBBR as well as the anaerobic and aerobic zones of the AAFBBR. The primary intermedia products in AFBBR (Supplementary Figure 1) were *p*-nitrosophenol (C_6_H_5_NO_2_), NC, HQ and PAP. Based on signal intensity, the relative concentrations followed the order: HQ > PAP > *p*-nitrosophenol > NC. The primary intermediate products in the anaerobic zone of the AAFBBR (Supplementary Figure 2) were PNP, *p*-hydroxyaminophenol (C_6_H_7_NO_2_), PAP and HQ. Based on signal intensity, the relative concentrations followed the order: HQ > PNP > PAP > *p*-hydroxyaminophenol. The primary intermediate products in the aerobic zone of the AAFBBR (Supplementary Figure 3) were PNP, PAP, HQ and NC. Based on signal intensity, the relative concentrations followed the order: HQ > PAP > PNP > NC.

**Figure 5:**
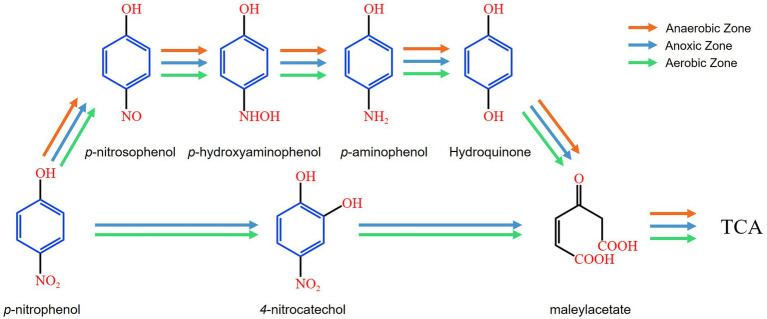
Intermediate products and proposed biodegradation pathways in the AFBBR and the AAFBBR.

The reduction of the nitro functional group (-NO_2_) to the amino functional group (-NH_2_) is generally considered to follow a stepwise process. Initially, the nitro group is reduced to a nitroso group (-NO), which is subsequently converted into a hydroxylamine group (-NHOH) (Mu et al., 2004). Finally, the hydroxylamine group undergoes further reduction to form the amino group (-NH_2_) (Mu et al., 2004). The detection of intermediate products, including p-nitrosophenol and p-hydroxyaminophenol, confirmed that PNP underwent stepwise reduction to PAP in the AFBBR and the AAFBBR. As shown in Figure 5, PNP was reduced to PAP and then hydroxyl-substituted amino groups were transformed under reductive conditions (Luo et al., 2021), leading to the formation of HQ. In addition, the NC was ovserved in the AFBBR and the aerobic zone of the AAFBBR, albeit at low concentrations, suggesting that the hydroxyquinol pathway occurred with the present of aerobic bacteria. Thus, the PNP biodegradation pathway in the AFBBR was inferred to involve both the hydroquinone and the hydroxyquinol pathways, with the hydroquinone pathway being the dominant route. In the anaerobic zone of the AAFBBR, PNP degradation primarily proceeded via the hydroquinone pathway, while biodegradation mechanisms in the aerobic zone of the AAFBBR closely resembled those observed in the AFBBR.

### 3.3 Microbial community analysis

The biodegradation mechanism of PNP was closely linked to the microbial communities in the AFBBR and the AAFBBR. To investigate the microbial community composition, biofilm samples were collected from the AFBBR and both the anaerobic and aerobic zones of AAFBBR at two critical stages: before the inoculate of PNP (Anoxic_day 0, Anaerobic_day 0 and Aerobic_day 0) and on the day 83 when PNP removal efficiency reached the maximum (Anoxic_day 83, Anaerobic_day 83 and Aerobic_day 83). These samples were subsequently analyzed using high-throughput sequencing. The number of sequences, the species richness (OTU abundances), and the Alpha diversity indices of all samples were listed in Table 2.

**Table 2:** Microbial richness and diversity in the AFBBR and the AAFBBR.

Samples	Number of sequences	Number of total OTUs	Alpha diversity indices			
			Coverage	Chao	Shannon	Simpson
Anoxic_day 0	32,795	371	0.996233	492.000000	3.175714	0.08016
Anoxic_day 83	50,911	343	0.996389	468.849057	3.293498	0.079518
Anaerobic_day 0	51,479	707	0.998339	847.123967	4.19701	0.046812
Anaerobic_day 83	50,077	334	0.997136	410.109091	3.130425	0.094015
Aerobic_day 0	34,049	763	0.995196	806.907563	4.478895	0.024908
Aerobic_day 83	45,783	386	0.996731	483.500000	3.525593	0.080666

**Figure 6:**
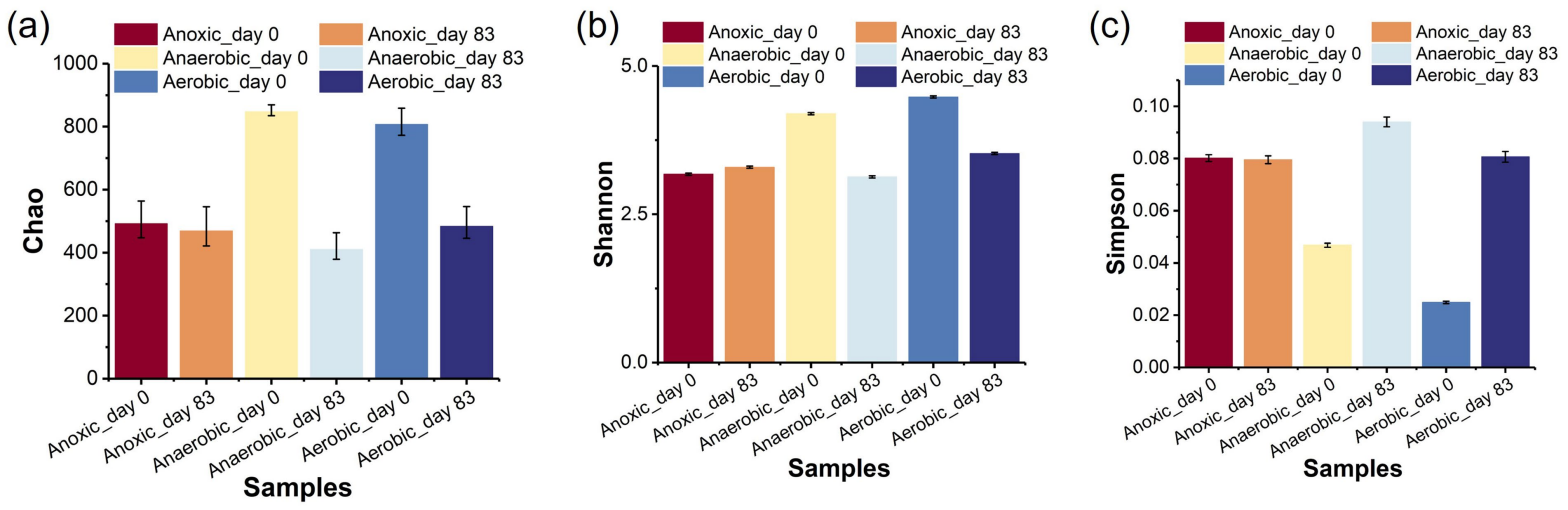
Analysis of Alpha diversity indices: **(a)** Chao, **(b)** Shannon, **(c)** Simpson.

The Alpha diversity analysis included Coverage, Chao, Simpson and Shannon indices. The Coverage indexes of all four samples exceeded 0.995, indicating that the sequencing results effectively reflected the true microbial composition of the samples. The Chao index was used to assess community richness, while the Shannon index measured microbial diversity and evenness. The Simpson index, on the other hand, estimated microbial dominance (Tong et al., 2022). Comparing the two operation stages, microbial diversity and richness showed clear changes with PNP exposure (Figure 4a). The Chao1 (Figure 6a) index was much higher in Anaerobic_day 0 and Aerobic_day 0 than in Anaerobic_day 83 and Aerobic_day 83, while Anoxic_day 0 had slightly higher value than Anoxic_day 83. This suggests that the increased PNP toxicity reduced microbial community richness, microorganisms unable to survive in the PNP environment were gradually removed from two reactors. In addition, the Shannon (Figure 6b) index was significantly higher in Anaerobic_day 0 and Aerobic_day 0 than that in Anaerobic_day 83 and Aerobic_day 83, while Anoxic_day 83 showed a marginal increase compared to Anoxic_ day 0. Conversely, Simpson index analysis (Figure 6c) revealed an inverse patterns: Anaerobic_day 0 and Aerobic_day 0 displayed lower values than Anaerobic_day 83 and Aerobic_day 83, whereas AFBBR maintained comparable values. These results indicate that as the PNP degradation system operated, microbial diversity decreased in both anaerobic and aerobic zones of the AAFBBR with a few PNP-tolerant and metabolizing bacteria becoming dominant. Since microbial pollutant removal is primarily driven by functional microbial groups, there was no direct correlation between PNP removal efficiency and community diversity (Tong et al., 2022). Instead, the dominance of PNP-degrading microorganisms was the key factor contributing to the enhanced PNP removal rate. However, the microbial community exhibited a slight increase in diversity while maintaining stable evenness, suggesting selective enrichment of specific functional taxa without disrupting the overall community balance. These results may be due to the anoxic environment led to the simultaneous growth of anaerobic, anoxic and aerobic microorganisms.

**Figure 7:**
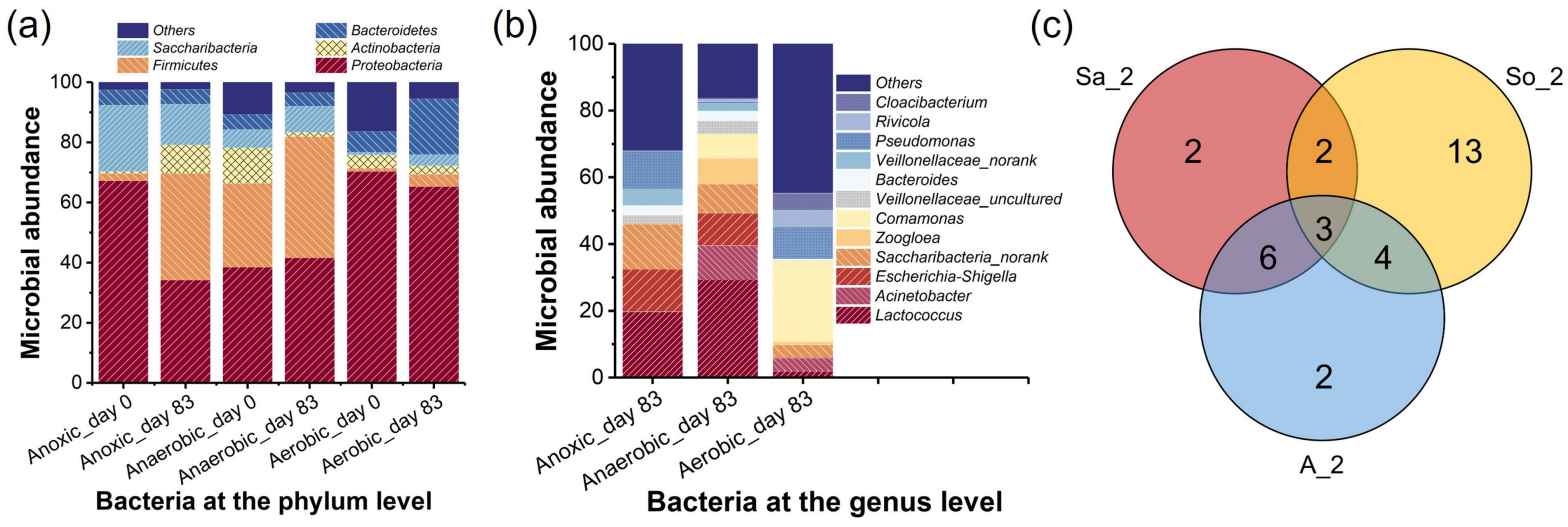
Microbial community analysis at the **(a)** phylum level, **(b)** genus level, and **(c)** Venn diagram.

The Venn diagram (Figure 7c) illustrates the shared and unique OTUs (relative abundance > 1.0%) in the AFBBR and the AAFBBR when PNP removal efficiency reached the maximum, providing an intuitive representation of the similarity and overlap in OTU composition. The bacterial composition and abundance were primarily affected by the introduction of PNP wastewater. Some bacterial populations declined, likely due to the inhibitory effects of PNP, while others showed a significant increase, demonstrating their strong adaptability and metabolic capacity in the PNP environment. There were three overlapping OTUs (*Saccharibacteria_norank*, *Enterobacter*, and *Lactococcus*) among all three samples. Six OTUs (*Bacteroides*, *Escherichia-Shigella*, *Veillonellaceae_uncultured*, *Streptococcus*, *Veillonella* and *Veillonellaceae_norank*) were shared between Anaerobic_day 83 and Anoxic_day 83, both of which were under low DO conditions. Additionally, two OTUs (*Comamonas*, *Acinetobacter*) were common between Anaerobic_day 83 and Aerobic_day 83, as both samples were collected from the AAFBBR. These findings suggest that these microbial taxa exhibit strong adaptability and may play a crucial role in PNP metabolism. The characteristics and functional roles of these bacteria will be further explored in the following sections.

The main taxonomic analysis in this experiment assigned to phylum and genus taxa level were summarized in Figure 7. The effects of the PNP introduction on biofilm community structure in the AFBBR and AAFBBR were compared at the phylum level, as well as the potential functional bacteria at the genus level were summarized. Within the AFBBR and the AAFBBR, distinct DO conditions were established across different areas, enabling the simultaneous coexistence and metabolic activity of anaerobic, anoxic, and aerobic microorganisms in both reactors.

The taxonomic classifications of effective bacterial sequences from biofilm samples before and after the introduction of PNP at the phylum level are summarized in Figure 7a, highlighting the effects of PNP wastewater on microbial community dynamics. Before the introduction of PNP wastewater, as shown in Figure 7a, there were mainly five phyla in the six samples, accounting for 97, 89 and 83% of the total microbial population in the Anoxic_day 0, Anaerobic_day 0 and Aerobic_day 0, respectively, including *Proteobacteria* (67% in Anoxic_day 0, 38% in Anaerobic_day 0, 70% in Aerobic_day 0), *Firmicutes* (2, 28, 1%), *Actinobacteria* (1, 12, 4%), *Saccharibacteria* (22, 6, 1%) and *Bacteroidetes* (5, 5, 7%). These phyla are commonly found in activated sludge microbial community (Liao et al., 2014), and the variations in microbial diversity and richness could be attributed to the different DO concentrations in anaerobic, anoxic and aerobic conditions. After 83 days of operations and optimizations, significant shifts in microbial richness were observed at the phylum level when the average PNP removal rate reached approximately 88.8 and 95.3% in the AFBBR and the AAFBBR, respectively. At this stage, the dominant phyla in the AFBBR (Anoxic_day 83) were *Proteobacteria* (34%), *Firmicutes* (36%), and Saccharibacteria (14%). In the AAFBBR, the dominant phyla in the anaerobic zone (Anaerobic_day 83) were *Proteobacteria* (42%), *Firmicutes* (40%), *Saccharibacteria* (9%), while *Proteobacteria* (65%), *Bacteroidetes* (19%), and *Firmicutes* (4%) were the dominant phyla in the aerobic zone (Aerobic_day 83). Among the six samples, *Proteobacteria* was the most abundant phylum before and after the introduction of PNP wastewater, indicating that *Proteobacteria* has strong adaptability to PNP wastewater. Ji et al. (2017) reported that *Proteobacteria* exhibit significant metabolic versatility in the biodegradation of phenolics, polycyclic aromatic hydrocarbons and heterocyclic, illustrating its potential metabolic capability to PNP. *Bacteroidetes* remained a high abundance in the AFBBR and AAFBBR, especially in the aerobic zone of the AFBBR, many species within this phylum are known to be responsible for denitrifying and organic pollutions degrading process (Yang et al., 2024). The high nitrogen removal efficiency observed in the AAFBBR could be attributable to the substantial abundance of *Bacteroidetes*. Additionally, with the introduce of PNP wastewater, *Firmicutes* exhibited a notable increase in anaerobic (27 to 40%) and aerobic zone (1 to 4%) of the AAFBBR and in the AFBBR (2 to 36%). This trend aligns with the ability of *Firmicutes* to form endospores, granting them high resistance to environmental stressors and enabling the efficient degradation of various recalcitrant organic compounds (Garcia et al., 2011).

*Actinobacteria*, which initially comprised a significant proportion in the AAFBBR (12% in the anaerobic and 4% in the aerobic zones), experienced a marked decline after 92 days (to 1 and 3%, respectively). This observation similar with the finding of Tong et al. (2022) and may attributed to the inhabitation of high influent PNP concentration. However, the abundance of *Actinobacteria* in AFBBR increased from 1 to 9%, suggesting its better adaptability in the anoxic condition. With the introduction of PNP wastewater, the relative abundance of *Saccharibacteria* increased in anaerobic zone (6 to 9%) and aerobic zone (1 to 4%) of the AAFBBR, while decreased in the AFBBR (22 to 14%). Prior studies have reported that *Saccharibacteria* possess metabolic potential for degrading complex carbon sources and play a role in biofilm formation (Li and Bae, 2024). Moreover, it was reported that *Saccharibacteria* had been identified as a key taxon in plastic degradation in polylactic acid soil environment (Ruthi et al., 2020). Under PNP-induced acclimatization, *Proteobacteria*, *Firmicutes*, *Actinobacteria*, *Saccharibacteria*, and *Bacteroidetes* exhibited strong adaptability to the high-toxicity PNP environment, sustaining growth and metabolic activity. Synergistic interactions among these diverse microbial communities played a crucial role in facilitating the efficient biodegradation of PNP under the anaerobic, anoxic, and aerobic conditions.

Figure 7b presents the taxonomic analysis at the genus level when the average PNP removal rate reached approximately 88.8 and 95.3% in the AFBBR and the AAFBBR, providing deeper insights into the potential functional bacteria present in two reactors. *Lactococcus* (20% in Anoxic_day 83 and 29% in Anaerobic_day 83), *Escherichia-Shigella* (13% in Anoxic_day 83 and 10% in Anaerobic_day 83), and *Saccharibacteria_norank* (14% in Anoxic_day 83 and 9% in Anaerobic_day 83) were the dominant genera in the AFBBR and the anaerobic zone of the AAFBBR, indicating their adaption and survival under low DO conditions with the inlet of PNP wastewater. Among these genera, *Lactococcus*, as a member of the phylum *Firmicutes*, is reported to play an important role in organic matter degradation and acidogenic fermentation (Cui et al., 2022; Wang C. et al., 2020). *Escherichia-Shigella* is reported to be associated with nitrogen removal (Sun et al., 2022) and *Saccharibacteria_norank* is associated with pyrimidine degradation (Hou et al., 2020), suggesting its potential involvement in organic compounds metabolism.

In addition, *Acinetobacter* (10% in Anaerobic_day 83 and 4% in Aerobic_day 83), *Comamonas* (7 and 24%), *Zoogloea* (8 and 1%), and *Pseudomonas* (1 and 10%) had high abundance in the AAFBBR. *Acinetobacter* has been reported to exhibit exceptional phenol degradation capability (Gallego et al., 2007), and *Comamonas* is known for its efficient metabolism of phenolic compounds (Huang et al., 2016; Tong et al., 2022). *Pseudomonas* is a typical gram-negative bacterium that is commonly enriched in the PNP wastewater treatment processes (Badamasi et al., 2025). Previous studies have demonstrated that members of the genus *Pseudomonas* could utilize PNP as their sole source of carbon, nitrogen, and energy (Chen et al., 2016), and are capable of degrading PNP via both the hydroquinone and hydroxyquinol pathways (Zhang et al., 2012). Moreover, *Zoogloea i*s an important component of activated sludge and biofilms, plays a vital role in the structure and performance of sludge flocs (Gao et al., 2014). The enrichment of *Zoogloea* is beneficial for enhancing the resistance of biofilm to adverse environmental conditions.

The aerobic zone of the AAFBBR also contained unique microbial genera, such as *Cloacibacterium* (5%), *Geobacter* (1%), and *Rivicola* (5%). Although their relative abundances were low, their roles in AAFBBR could not be overlooked. Studies have reported that *Cloacibacterium* could utilize biodegradable organic matter as a carbon source to produce EPS, which protect the bacteria from the toxicity of high concentrations of recalcitrant organic pollutants (Ram et al., 2018). *Geobacter*, on the other hand, has demonstrated the capability to oxidize benzene-derived and phenolic compounds (Zhou et al., 2016), suggesting its potential role in PNP degradation. Additionally, *Rivicola* has been reported to participate in nitrogen removal (Huang et al., 2024) and its increased abundance may be attributed to nitrogen supplement in AAFBBR.

Building on the conclusions in section 3.2, the PNP biodegradation pathway was inferred to involve both the hydroquinone and the hydroxyquinol pathways, which can be attributed to the synergistic interactions among diverse microbial communities under varying DO conditions. In both the AFBBR and the AAFBBR, key bacterial genera, including *Lactococcus*, *Escherichia-Shigella*, *Saccharibacteria_norank*, *Acinetobacter*, *Comamonas*, *Zoogloea*, and *Pseudomonas*, were suggested to play crucial roles in the hydroquinone pathway. Notably, the hydroxyquinol pathway was observed only in the AFBBR and the aerobic zone of the AAFBBR, where the DO concentrations were higher. *Pseudomonas*, a key microorganism capable of degrading PNP via both the hydroquinone and hydroxyquinol pathways, exhibited significantly higher abundance in the anoxic and aerobic zones than in the anaerobic zone of the AAFBBR. This suggests that *Pseudomonas* was likely a major contributor to the hydroxyquinol degradation pathway.

## 4 Conclusion

This study successfully established an anoxic fluidized bed bioreactor (AFBBR) and an anaerobic-aerobic fluidized bed bioreactor (AAFBBR) for enhancing PNP biodegradation efficiency during 90-day operation. Under an influent PNP concentration of 100 mg/L, a glucose to PNP co-substrate ratio of 6:1, and a C/N ratio of 10:1, the AFBBR achieved PNP, COD, and TN removal efficiencies of 88.8 ± 1.0%, 80.9 ± 8.9%, and 47.0 ± 3.1%, respectively, while the AAFBBR reached 95.3 ± 0.3%, 90.4 ± 0.7%, and 61.9 ± 4.2%, respectively. PNP biodegradation in both reactors followed the hydroquinone and hydroxyquinol pathways, with the hydroquinone pathway being dominant. *Proteobacteria* (34% in the AFBBR, 42 and 65% in the anaerobic and aerobic zones of the AAFBBR), *Firmicutes* (35, 40, and 4%), *Saccharibacteria* (14, 9, and 4%) and *Bacteroidetes* (5, 4 and 19%) were the predominant phyla in PNP biodegradation. Key bacterial genera, including *Lactococcus*, *Escherichia-Shigella*, *Saccharibacteria_norank*, *Acinetobacter*, *Comamonas*, *Zoogloea*, and *Pseudomonas*, played crucial roles in the hydroquinone pathway, with *Pseudomonas* as the key contributor to the hydroxyquinol pathway. These findings provide a scientific foundation and theoretical support for the pilot-scale implementation and efficient biological treatment of PNP in the industrial and agricultural wastewater.

## Data Availability

The data presented in the study are deposited in the NCBI repository, accession number PRJNA1258386.

## References

[ref1] Badamasi H. Naeem Z. Antoniolli G. Kumar A. P. Olaleye A. A. Sadiq I. S.. (2025). A review of recent advances in green and sustainable technologies for removing *4*-nitrophenol from the water and wastewater. Sustain. Chem. Pharm. 43:101867. doi: 10.1016/j.scp.2024.101867, PMID: 40266665

[ref2] Bai Q. Yang L. Li R. Chen B. Zhang L. Zhang Y.. (2015). Accelerating quinoline biodegradation and oxidation with endogenous electron donors. Environ. Sci. Technol. 49, 11536–11542. doi: 10.1021/acs.est.5b03293, PMID: 26327306

[ref3] Chen Q. Tu H. Luo X. Zhang B. Huang F. Li Z.. (2016). The regulation of *Para*-Nitrophenol degradation in *Pseudomonas putida* DLL-E4. PLoS One 11:18. doi: 10.1371/journal.pone.0155485, PMID: 27191401 PMC4871426

[ref4] Chowdhury M. Nakhla G. (2022). Enhanced mainstream nitrogen removal from synthetic wastewater using gel-immobilized anammox in fluidized bed bioreactors: process performance and disintegration mechanisms. Sci. Total Environ. 811:151373. doi: 10.1016/j.scitotenv.2021.151373, PMID: 34748847

[ref5] Cui Y. Zhang H. Zhang J. Lv B. Xie B. (2022). The emission of volatile organic compounds during the initial decomposition stage of food waste and its relationship with the bacterial community. Environ. Technol. Innov. 27:102443. doi: 10.1016/j.eti.2022.102443, PMID: 40266665

[ref6] Eldyasti A. Chowdhury N. Nakhla G. Zhu J. (2010). Biological nutrient removal from leachate using a pilot liquid-solid circulating fluidized bed bioreactor (LSCFB). J. Hazard. Mater. 181, 289–297. doi: 10.1016/j.jhazmat.2010.05.010, PMID: 20510504

[ref7] Feng A. Lin C. Zhou H. Jin W. Hu Y. Li D.. (2024). Catalytic transformation of *4*-nitrophenol into *4*-aminophenol over ZnO nanowire array-decorated cu nanoparticles. Green Chem. Eng. 5:205212, 205–212. doi: 10.1016/j.gce.2023.03.003

[ref8] Gallego J. L. R. García-Martínez M. J. Llamas J. F. (2007). Biodegradation of oil tank bottom sludge using microbial consortia. Biodegradation 18:269281, 269–281. doi: 10.1007/s10532-006-9061-y, PMID: 16821101

[ref9] Gao C. Wang A. Wu W. Yin Y. Zhao Y. (2014). Enrichment of anodic biofilm inoculated with anaerobic or aerobic sludge in single chambered air-cathode microbial fuel cells. Bioresour. Technol. 167:124132, 124–132. doi: 10.1016/j.biortech.2014.05.120, PMID: 24973773

[ref10] Garcia S. L. Jangid K. Whitman W. B. Das K. C. (2011). Transition of microbial communities during the adaption to anaerobic digestion of carrot waste. Bioresour. Technol. 102, 7249–7256. doi: 10.1016/j.biortech.2011.04.098, PMID: 21620691

[ref11] Hou H. Duan L. Zhou B. Tian Y. Wei J. Qian F. (2020). The performance and degradation mechanism of sulfamethazine from wastewater using IFAS-MBR. Chinese Chem. Lett. 31:543546, 543–546. doi: 10.1016/j.cclet.2019.08.031

[ref12] Hu F. Zhou H. Jin Z. Sun Q. Pan Z. Zhu J. (2013). Biodegradation of TCP in a sequencing batch-fluidized bed bioreactor with waste coke particles as the carrier. J. Environ. Eng. 139, 1222–1227. doi: 10.1061/(ASCE)EE.1943-7870.0000728

[ref13] Huang K. He Y. Wang W. Jiang R. Zhang Y. Li J.. (2024). Temporal differentiation in the adaptation of functional bacteria to low-temperature stress in partial denitrification and anammox system. Environ. Res. 244:117933. doi: 10.1016/j.envres.2023.117933, PMID: 38097061

[ref14] Huang Y. Hou X. Liu S. Ni J. (2016). Correspondence analysis of bio-refractory compounds degradation and microbiological community distribution in anaerobic filter for coking wastewater treatment. Chem. Eng. J. 304:864872, 864–872. doi: 10.1016/j.cej.2016.05.142

[ref15] Ji F. Yuan Y. Lai B. (2017). Microbial community dynamics in aerated biological fluidized bed (ABFB) with continuously increased *p*-nitrophenol loads. Process Biochem. 63, 185–192. doi: 10.1016/j.procbio.2017.07.033

[ref16] Karadag D. Koeroglu O. E. K. Ozkaya B. Cakmakci M. (2015). A review on anaerobic biofilm reactors for the treatment of dairy industry wastewater. Process Biochem. 50, 262–271. doi: 10.1016/j.procbio.2014.11.005

[ref7001] Kitagawa W. Kimura N. Kamagata Y. (2004). A novel p-nitrophenol degradation gene cluster from a gram-positive bacterium, Rhodococcus opacus SAO101. J. Bacteriol. 186, 4898–4902. doi: 10.1128/JB.186.15.4894-4902.2004 PMC451640 15262926

[ref17] Kuyukina M. S. Ivshina I. B. Serebrennikova M. K. Krivoruchko A. V. Korshunova I. O. Peshkur T. A.. (2017). Oilfield wastewater biotreatment in a fluidized-bed bioreactor using co-immobilized *Rhodococcus* cultures. J. Environ. Chem. Eng. 5, 1252–1260. doi: 10.1016/j.jece.2017.01.043

[ref18] Li M. Bae S. (2024). Exploring the effects of polyethylene and polyester microplastics on biofilm formation, membrane fouling, and microbial communities in modified ludzack-ettinger-reciprocation membrane bioreactors. Bioresour. Technol. 414, 0960–8524. doi: 10.1016/j.biortech.2024.131636, PMID: 39414168

[ref19] Liao R. Li Y. Yu X. Shi P. Wang Z. Shen K.. (2014). Performance and microbial diversity of an expanded granular sludge bed reactor for high sulfate and nitrate waste brine treatment. J. Environ. Sci. 26:717725, 717–725. doi: 10.1016/S1001-0742(13)60479-9, PMID: 25079401

[ref20] Liu X. Kim M. Nakhla G. Andalib M. Fang Y. (2020). Partial nitrification-reactor configurations, and operational conditions: performance analysis. J. Environ. Chem. Eng. 8:103984. doi: 10.1016/j.jece.2020.103984

[ref21] Lu R. Hong B. Wang Y. Cui X. Liu C. Liu Y.. (2025). Microalgal biofilm cultivation on lignocellulosic based bio-carriers: effects of material physical characteristics on microalgal biomass production and composition. Chem. Eng. J. 510:161656. doi: 10.1016/j.cej.2025.161656

[ref22] Luo J. Xu Y. Wang J. Zhang L. Jiang X. Shen J. (2021). Coupled biodegradation of *p*-nitrophenol and *p*-aminophenol in bioelectrochemical system: mechanism and microbial functional diversity. J. Environ. Sci. 108, 134–144. doi: 10.1016/j.jes.2021.02.017, PMID: 34465427

[ref23] Mallikarjuna C. Dash R. R. (2020). A review on hydrodynamic parameters and biofilm characteristics of inverse fluidized bed bioreactors for treating industrial wastewater. J. Environ. Chem. Eng. 8:104233. doi: 10.1016/j.jece.2020.104233

[ref24] Mei X. Liu J. Guo Z. Li P. Bi S. Wang Y.. (2019). Simultaneous *p*-nitrophenol and nitrogen removal in PNP wastewater treatment: comparison of two integrated membrane-aerated bioreactor systems. J. Hazard. Mater. 363, 99–108. doi: 10.1016/j.jhazmat.2018.09.072, PMID: 30308370

[ref25] Mu Y. Yu H. Zheng J. Zhang S. Sheng G. (2004). Reductive degradation of nitrobenzene in aqueous solution by zero-valent iron. Chemosphere 54, 789–794. doi: 10.1016/j.chemosphere.2003.10.023, PMID: 14637335

[ref26] Ram S. K. Kumar L. R. Tyagi R. D. Drogui P. (2018). Techno-economic evaluation of simultaneous production of extra-cellular polymeric substance (EPS) and lipids by *Cloacibacterium normanense* NK6 using crude glycerol and sludge as substrate. Water Sci. Technol. 77, 2228–2241. doi: 10.2166/wst.2018.140, PMID: 29757175

[ref7002] Ruthi J. Blsterli D. Pardi Comensoli L. Brunner I. Frey B. (2020). The “Plastisphere” of biodegradable plastics is characterized by specific microbial taxa of alpine and arctic soils. Env. Sci. 8:562263. doi: 10.3389/fenvs.2020.562263, PMID: 14637335

[ref27] Sponza D. T. Kuscu Z. S. (2011). Relationships between acute toxicities of *Para* nitrophenol (*p*-NP) and nitrobenzene (NB) to *Daphnia magna* and *Photobacterium phosphoreum*: physicochemical properties and metabolites under anaerobic/aerobic sequentials. J. Hazard. Mater. 185, 1187–1197. doi: 10.1016/j.jhazmat.2010.10.030, PMID: 21035948

[ref28] Sun C. Li C. Zhang K. Ma X. Zhang Y. (2022). Six complex microbial inoculants for removing ammonia nitrogen from waters. Water Environ. Res. 94:e10823. doi: 10.1002/wer.10823, PMID: 36544243

[ref29] Sun J. Yuan M. Zhou H. Chen Z. Wang Y. Cheng H. (2025). Enhanced printing and dyeing wastewater treatment using anaerobic-aerobic systems with bioaugmentation. J. Hazard. Mater. 486:136982. doi: 10.1016/j.jhazmat.2024.136982, PMID: 39729804

[ref30] Tang P. Deng C. Tang X. Si S. Xiao K. (2012). Degradation of *p*-nitrophenol by interior microelectrolysis of zero-valent iron/copper-coated magnetic carbon galvanic couples in the intermittent magnetic field. Chem. Eng. J. 210, 203–211. doi: 10.1016/j.cej.2012.08.089

[ref31] Tong J. Cui L. Wang D. Wang X. Liu Z. (2022). Assessing the performance and microbial structure of biofilms in membrane aerated biofilm reactor for high *p*-nitrophenol concentration treatment. J. Environ. Chem. Eng. 10:108635. doi: 10.1016/j.jece.2022.108635

[ref32] Vo H. N. P. Ngo H. H. Guo W. Liu Y. Chang S. W. Nguyen D. D.. (2020). Selective carbon sources and salinities enhance enzymes and extracellular polymeric substances extrusion of *Chlorella* sp. for potential co-metabolism. Bioresour. Technol. 303:122877. doi: 10.1016/j.biortech.2020.122877, PMID: 32028214

[ref33] Wang H. He X. Nakhla G. Zhu J. Su Y. (2020). Performance and bacterial community structure of a novel inverse fluidized bed bioreactor (IFBBR) treating synthetic municipal wastewater. Sci. Total Environ. 718:137288. doi: 10.1016/j.scitotenv.2020.137288, PMID: 32087585

[ref34] Wang L. Hu Z. Hu M. Zhao J. Zhou P. Zhang Y.. (2022). Cometabolic biodegradation system employed subculturing photosynthetic bacteria: a new degradation pathway of *4*-chlorophenol in hypersaline wastewater. Bioresour. Technol. 361:127670. doi: 10.1016/j.biortech.2022.127670 35878775

[ref35] Wang J. Wang D. Su Z. Song Y. Zhang J. Xiahou Y. (2024). Green synthesis of chitosan/glutamic acid/agarose/ag nanocomposite hydrogel as a new platform for colorimetric detection of cu ions and reduction of *4*-nitrophenol. Int. J. Biol. Macromol. 259:129394. doi: 10.1016/j.ijbiomac.2024.129394, PMID: 38218277

[ref36] Wang C. Yu G. Yang F. Wang J. (2020). Formation of anaerobic granules and microbial community structure analysis in anaerobic hydrolysis denitrification reactor. Sci. Total Environ. 737:139734. doi: 10.1016/j.scitotenv.2020.139734, PMID: 32526572

[ref37] Xu J. Wang B. Zhang W. Zhang F. Deng Y. Wang Y.. (2021). Biodegradation of *p*-nitrophenol by engineered strain. AMB Expr. 11:124. doi: 10.1186/s13568-021-01284-8, PMID: 34463855 PMC8408293

[ref38] Yan H. Gu Z. Zhang Q. Wang Y. Cui X. Liu Y.. (2024). Detoxification of copper and zinc from anaerobic digestate effluent by indigenous bacteria: mechanisms, pathways and metagenomic analysis. J. Hazard. Mater. 469:133993. doi: 10.1016/j.jhazmat.2024.133993 38461661

[ref39] Yan H. Lu R. Liu Y. Cui X. Wang Y. Yu Z.. (2022). Development of microalgae-bacteria symbiosis system for enhanced treatment of biogas slurry. Bioresour. Technol. 354:127187. doi: 10.1016/j.biortech.2022.127187, PMID: 35439556

[ref40] Yang X. Liao Y. Zeng M. Qin Y. (2024). Nitrite accumulation performance and microbial community of algal-bacterial symbiotic system constructed by *Chlorella* sp. and *Navicula* sp. Bioresour. Technol. 399:130638. doi: 10.1016/j.biortech.2024.130638, PMID: 38548030

[ref41] Ye F. Ye Y. Li Y. (2011). Effect of C/N ratio on extracellular polymeric substances (EPS) and physicochemical properties of activated sludge flocs. J. Hazard. Mater. 188, 37–43. doi: 10.1016/j.jhazmat.2011.01.043, PMID: 21333444

[ref42] Zhang S. Sun W. Xu L. Zheng X. Chu X. Tian J.. (2012). Identification of the *Para*-nitrophenol catabolic pathway, and characterization of three enzymes involved in the hydroquinone pathway, in *pseudomonas* sp. 1-7. BMC Microbiol. 12, 1471–2180. doi: 10.1186/1471-2180-12-27, PMID: 22380602 PMC3324391

[ref43] Zheng M. Bai Y. Han H. Zhang Z. Xu C. Ma W.. (2021). Robust removal of phenolic compounds from coal pyrolysis wastewater using anoxic carbon-based fluidized bed reactor. J. Clean. Prod. 280:124451. doi: 10.1016/j.jclepro.2020.124451

[ref44] Zhou L. Deng D. Zhang D. Chen Q. Kang J. Fan N.. (2016). Microbial electricity generation and isolation of exoelectrogenic bacteria based on petroleum hydrocarbon-contaminated soil. Electroanalysis 28, 1510–1516. doi: 10.1002/elan.201501052

