# Supplementary material for: Biodegradation mechanisms of p-nitrophenol and microflora dynamics in fluidized bed bioreactors
Source: Front Microbiol. 2025 May 14;16:1602768. doi: 10.3389/fmicb.2025.1602768 (PMC12116535; doi:10.3389/fmicb.2025.1602768)
Supplement: Supplementary file 1 [file Data_Sheet_1.docx]

Supplementary Material

# Supplementary Figures and Tables

## Supplementary Figures


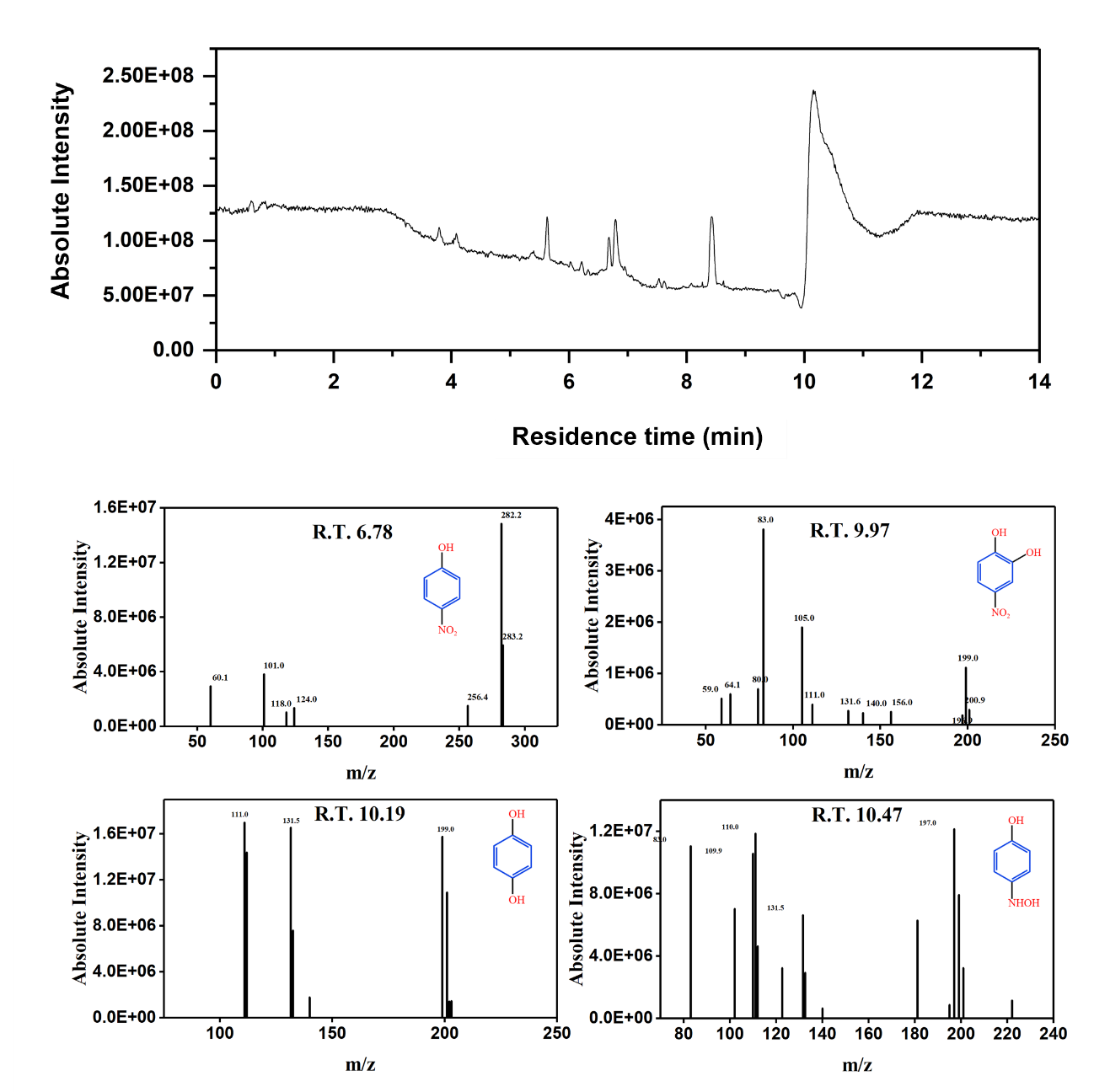


**Supplementary Figure 1.** primary intermedia products in AFBBR


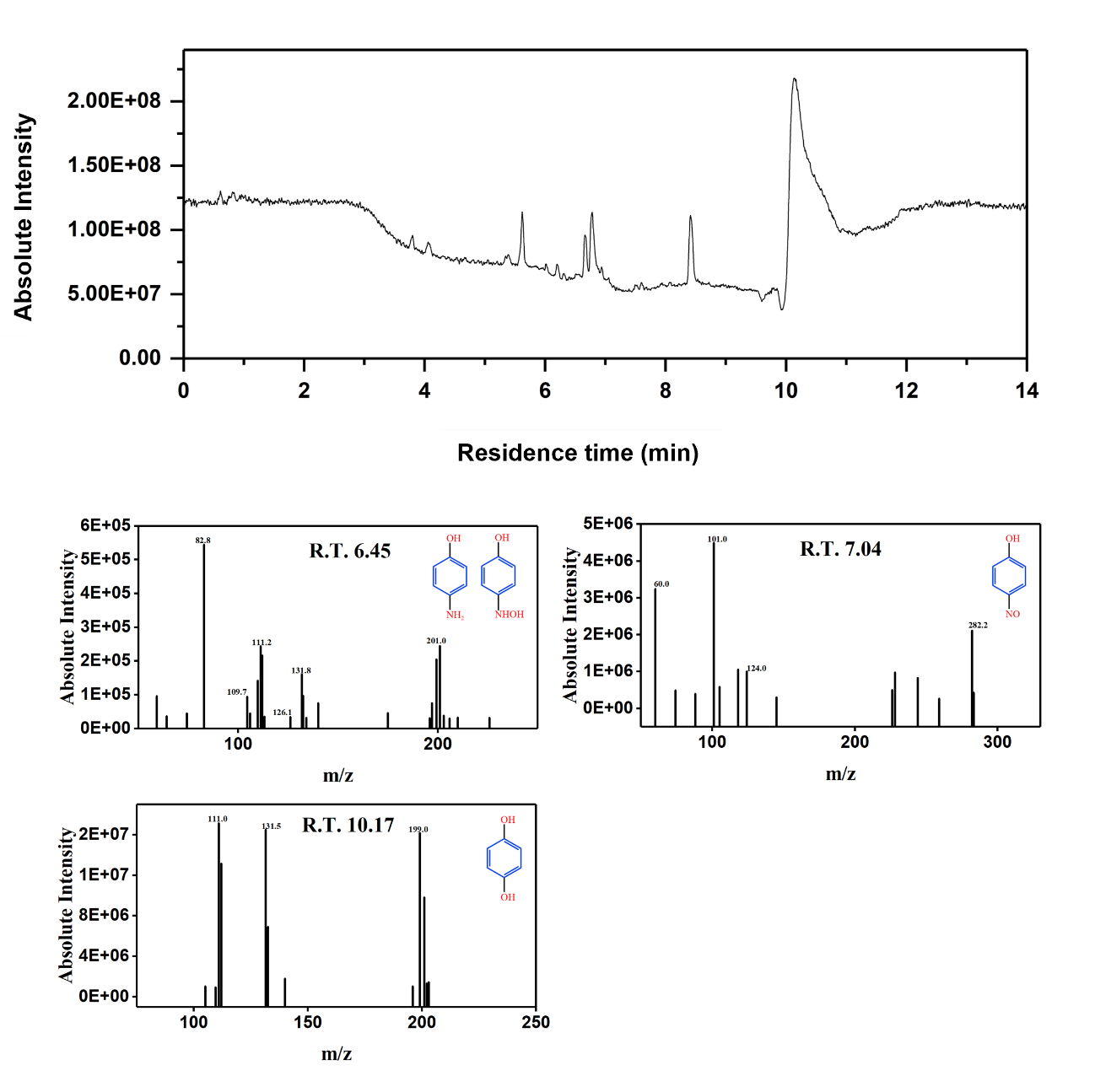
**Supplementary Figure 2.** primary intermediate products in the anaerobic zone of the AAFBBR


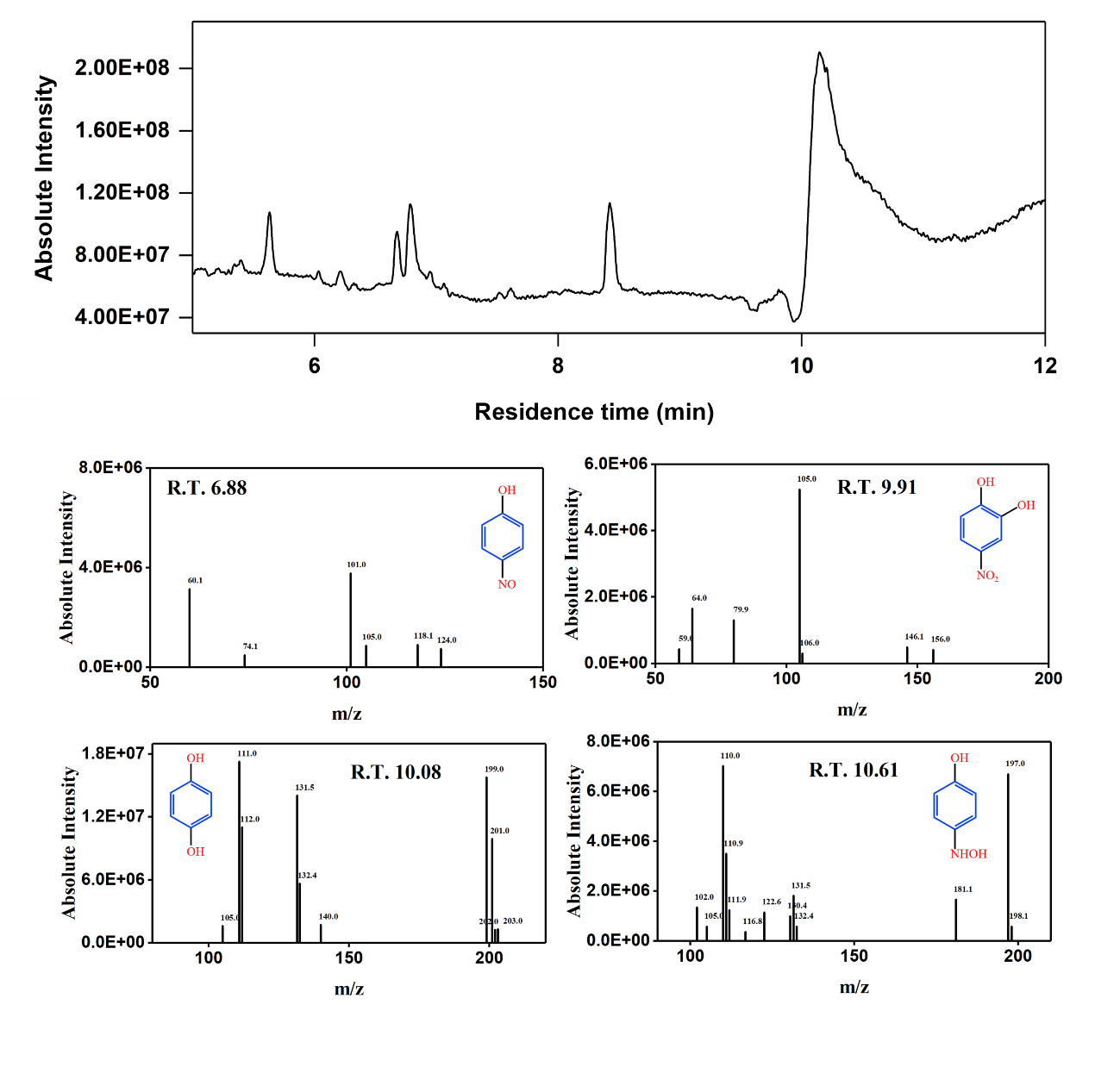
**Supplementary Figure 3.** primary intermediate products in the aerobic zone of the AAFBBR

## Supplementary Tables

**Supplementary Table 1.** The particle characteristics of the WCPs

| Paricle size  (mm) | True density  (kg/m^3^) | Bulk density  (kg/m^3^) | Porosity  (%) | Water absorption  (%) | Specific surface area  (m^2^/g) |
| --- | --- | --- | --- | --- | --- |
| 0.6~1.2 | 1598 | 675 | 57.7 | 41.2 | 55.9 |

**Supplementary Table 2.** The nutrient solution ratio table during the start-up

| **Chemicals** | **Concentration (mg/L)** |
| --- | --- |
| C_6_H_12_O_6_ | - |
| NH_4_Cl | 0 |
| KH_2_PO_4_ | 9 |
| FeCl_3_·3H_2_O | 0.1 |
| ZnCl | 0.05 |
| MnSO_4_·H_2_O | 0.06 |
| MgCl_2_ | 22 |
| FeSO_4_·7H_2_O | 1.5 |
| CaCl_2_·2H_2_O | 20 |
| Ni(CH_3_COO)_2_·4H_2_O | 0.02 |
| (NH_4_)Mo_2_O_24_·4H_2_O | 0.03 |
| CuCl_2_·2H_2_O | 0.05 |
| CoCl_2_·6H_2_O | 0.2 |

**Supplementary Table 3.** Main intermediate products in AABFBR

|  |  | No. | Retention time/min | m/z | Molecular formula | Name |
| --- | --- | --- | --- | --- | --- | --- |
| AFBBR | | 1 | 6.78 | 124 | C_6_H_5_NO_2_ | *p*-nitrosophenol |
|  |  | 2 | 9.97 | 156 | C_6_H_5_NO_4_ | 4-nitrocatechol |
|  |  | 3 | 10.19 | 111 | C_6_H_6_O_2_ | Hydroquinone |
|  |  | 4 | 10.47 | 110 | C_6_H_7_NO | *p*-aminophenol |
| AAFBBR | Anoxic Zone | 1 | 6.45 | 110 | C_6_H_7_NO | *p*-aminophenol |
|  |  | 2 | 6.45 | 126 | C_6_H_7_NO_2_ | *p*-hydroxyaminophenol |
|  |  | 3 | 7.05 | 124 | C_6_H_5_NO_2_ | *p*-nitrosophenol |
|  |  | 4 | 10.17 | 111 | C_6_H_6_O_2_ | Hydroquinone |
|  | Aerobic Zone | 1 | 6.89 | 124 | C_6_H_5_NO_2_ | *p*-nitrosophenol |
|  |  | 2 | 9.92 | 156 | C_6_H_5_NO_4_ | 4-nitrocatechol |
|  |  | 3 | 10.12 | 111 | C_6_H_6_O_2_ | Hydroquinone |
|  |  | 4 | 10.62 | 110 | C_6_H_7_NO | *p*-aminophenol |
